# CD11a regulates hematopoietic stem and progenitor cells

**DOI:** 10.3389/fimmu.2023.1219953

**Published:** 2023-09-14

**Authors:** Lifei Hou, Koichi Yuki

**Affiliations:** ^1^ Department of Anesthesiology, Critical Care and Pain Medicine, Cardiac Anesthesia Division, Boston Children’s Hospital, Boston, MA, United States; ^2^ Department of Anaesthesia, Harvard Medical School, Boston, MA, United States; ^3^ Department of Immunology, Harvard Medical School, Boston, MA, United States; ^4^ Broad Institute of Harvard and Massachusetts Institute of Technology (MIT), Cambridge, MA, United States

**Keywords:** CD11a, sepsis, hematopoietic stem and progenitor cells (HPSCs), il-27, inflammation

## Abstract

Integrin αLβ2 (CD11a/CD18, CD11a) is a critical leukocyte adhesion molecule in leukocyte arrest and immunological synapse formation. However, its role in the bone marrow has not been investigated in depth. Here we showed that CD11a was expressed on all subsets of hematopoietic stem and progenitor cells (HPSCs). CD11a deficiency enhanced HSPCs activity under lipopolysaccharide (LPS) stimulation as demonstrated by a higher HSPC cell count along with an increase in cell proliferation. However, our mixed chimera experiment did not support that this phenotype was driven in a cell-intrinsic manner. Rather we found that the production of IL-27, a major cytokine that drives HSPC proliferation, was significantly upregulated both *in vivo* and *in vitro*. This adds a novel role of CD11a biology.

## Introduction

Within the bone marrow (BM), hematopoietic stem and progenitor cells (HSPCs) reside in microenvironments called the stem cell niches (1, 2). HSPCs can interact with the cellular and non-cellular components at the niche via adhesion molecule family such as integrins (3).

Integrins are heterodimeric transmembrane molecules consisting of non-covalently linked α- and β- subunits. There are 18 α- and 8 β-subunits identified, creating 24 distinct integrins (4). Among them, β2 integrins are expressed exclusively on leukocytes and consist of four members αLβ2 (CD11a/CD18, later we call CD11a), αMβ2 (CD11b/CD18), αXβ2 (CD11c/CD18) and αDβ2 (CD11d/CD18). Different from other β2 integrin members, CD11a is ubiquitously expressed on all leukocytes in the periphery and plays a number of critical roles including leukocyte arrest and immunological synapse formation (5, 6). The expression of CD11a on bone marrow cells was previously reported (7), but its detailed expression profile as well as its functional role in the bone marrow are not delineated yet.

In this study, we examined the role of CD11a in HSPCs. We found that CD11a was expressed on all subsets of HPSCs, and its deficiency increased HPSC proliferation and its cell number upon lipopolysaccharide (LPS) stimulation. We also examined the mechanism.

## Methods

### Mice

CD11a KO, CD11b KO, CD11d KO, CD45.1 and CD45.2 wild type mice were purchased from the Jackson laboratory. CD11c KO mice were kindly given by Dr. Christine Ballantyne (Baylor University), as we previously described in our publication (8–10). Intercellular adhesion molecule-1 (ICAM-1) KO mice were kindly given by Dr. Gregory Priebe (Boston Children’s Hospital) (11). They were housed under specific pathogen free condition, with 12-hour light and dark cycles. All animal protocols were approved by the Institutional Animal Care and Use Committee (IACUC) at Boston Children’s Hospital.

### Flow cytometry analysis of hematopoietic stem and progenitor cells

Whole bone marrow cells were isolated from mice and stained on ice with various antibody cocktails to identify HSPC compartment. As we previously described (9), the lineage marker includes anti-mCD11b (M1/70), anti-mGr1 (RB6-8C5), anti-mCD3 (145-2C11), anti-CD4 (RM4-5), anti-CD8 (53-6.7), anti-mB220 (RA3-6B2), anti-mNK1.1 (PK136), and anti-mTer119 (TER-119). The other mouse antibodies include anti-mCD45.1 (A20), anti-CD45.2 (104), anti-mIgM (R6-60.2), anti-mC-kit (2B8), anti-mSca-1 (D7), anti-mCD34 (RAM34), anti-mCD150 (TC15-12F12.2), anti-mCD48 (HM48-1), anti-mIL7Rα (A7R34), and anti-mFcR (93), and its isotype control hamster IgG (A19-3). Antibodies were purchased from Biolegend (San Diego, CA) and BD Biosciences (Franklin Lakes, NJ). Cell counting was done by applying Sphero AccuCount beads (ACBP-50; Spherotech Inc, Lake Forest, IL). Data were acquired on a Canto II cytometer (BD Biosciences) and analyzed using FlowJo software (flowJo LLC, Ashland, OR).

### Proliferation assay

Cell proliferation was examined either with Ki67 staining or 5-bromo-2’-deoxyruridine (BrdU) staining. For Ki67, cells were subjected to intracellular staining using anti-mKi67 antibody (11F6, Biolegend). For BrdU incorporation, mice were injected intraperitoneally (*i.p*.) with 1 mg of BrdU. To detect BrdU incorporation into bone marrow hematopoietic cells, BD cytofix/cytoper Plus kit (555028, BD Biosciences) and Alexa fluor 488-conjugated anti-BrdU antibody (3D4, Biolegend) were applied according to the manufacturer’s protocol. Then cells were subjected to flow cytometry analysis.

### Systemic lipopolysaccharide injection model

Mice were subjected to lipopolysaccharide (LPS, from Pseudomonas aeruginosa 10, 10 mg/kg; Sigma, St. Louis, MO) injection via tail vein. Mice were euthanized at indicated time points for analysis.

### Chimera mouse experiments

To generate mixed bone marrow chimeras, recipient mice on the C57BL/6 background were irradiated with 2 doses of 550 rad with 4-hour intervals. Wild-type (WT, CD45.1) and WT (CD45.2) derived bone marrow cells at WT (CD45.1) and CD11a KO (CD45.2) derived bone marrow cells were mixed at the ratio of 1:1 with total of 5 x 10^6^ cells and intravenously (*i.v.*) injected into the tail vein of lethally irradiated recipients. Mice were evaluated for the reconstitution of the immune compartment at various time points after bone marrow transplantation. To prevent bacterial infection, the mice were provided with autoclaved drinking water containing sulfatrim for 1 week prior to and for 4 weeks after irradiation.

### Plasma or tissue culture medium cytokine measurement

Plasma IL-27 level or IL-27 levels in cell culture supernatant were measured using IL-27 ELISA kit per the company’s protocol (Thermo Fischer). TNF-α, KC, IL-1β, IL-6, IL-10, IL-12p70, and IFNγ in plasma or cell culture supernatant were measured using mouse cytokine 7-plex (Mesoscale, Rockville, MD) per the company protocol.

### Bone marrow derived macrophage and LPS stimulation

Bone marrow cells were isolated and red blood cells were lyzed. Single cell suspension was cultured in DMEM medium containing 10% FCS and recombinant murine M-CSF (rmM-CSF, Peprotech, 100 unit/ml) for 3 days. After equal volume of fresh medium containing 10% FCS and rmM-CSF (100 unit/ml) was added, cells were cultured for additional 4 days. At the end of differentiation, supernatant and non-adherent cells were discarded. The adherent cells were deemed as bone marrow-derived macrophage (BMDM) and used for further experiments. Regarding LPS stimulation, BMDMs were cultured with medium or LPS (1 µg/ml) for 24 hours. In some experiment, we used CD11a inhibitor GSK2344307A and GSK2344343A (10 µM) (12).

### Statistical analysis

Data were analyzed as indicated in the figure legend. Statistical significance was defined as P< 0.05. All the statistical calculations were performed using PRISM5 software (GraphdPad Software, La Jolla, CA).

## Results

### CD11a was expressed on HSPCs

**Figure 1:**
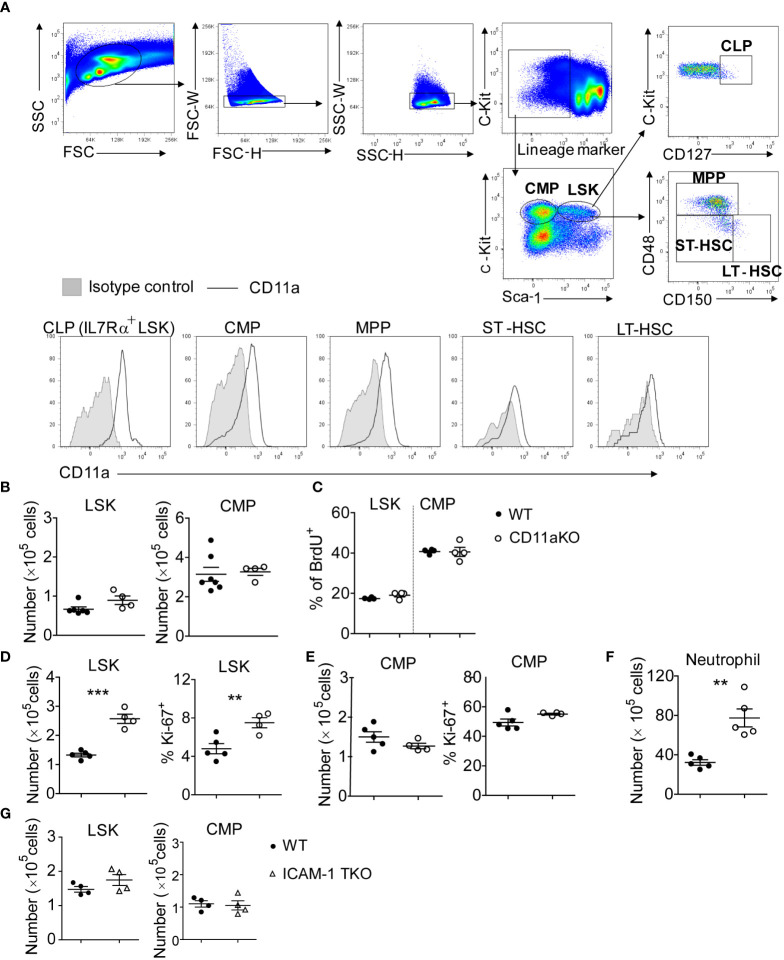
CD11a KO LSK cells proliferate robustly upon LPS injection. **(A)** Upper-panel: gating strategy by staining the WT bone marrows; bottom-panel: flow cytometry histogram showing the expression of CD11a on indicated populations. Shown are representative flow cytometry plots from one of three independent experiments. **(B)** BM LSK and CMP counted from naïve WT and CD11a KO mice. **(C)** Cumulative analysis of frequency of BrdU+ population in WT and CD11a KO mice. **(D–G)**. Mice were *i.v.* injected with LPS for 6 hours. **(D)** Left: absolute number of LSKs; Right: Ki67 expression detected intracellular staining and by flow cytometry; **(E)** Left: absolute number of CMPs; Right: Ki67 expression detected intracellular staining and by flow cytometry; **(F)** BM neutrophil counted from WT and CD11a KO mice upon LPS injection; **(G)** BM LSK and CMP counted from WT and ICAM1-KO mice (also named total KO, TKO) upon LPS injection. For B to G, symbols indicate individual mice. Data are representative of 2 experiments. Error bars indicate ± standard error of the mean (SEM). Statistical significance was tested with the Student t test. **P <0.01; ***P < 0.001.

Because CD11a is known to be expressed ubiquitously on peripheral leukocytes, we checked to see if CD11a was also expressed on hematopoietic stem and progenitor cells (HSPCs). We found that CD11a was expressed on all of long-term hematopoietic stem cells (LT-HSCs), short-term hematopoietic stem cells (ST-HSCs), multipotent progenitors (MPPs), common myeloid progenitors (CMPs) and common lymphoid progenitors (CLPs) (**Figure 1A**). The number of lineage^−^sca-1^+^c-kit^+^ (LSK) cells and CMPs was comparable between WT and CD11a KO mice (**Figure 1B**). No difference in proliferation was observed (**Figure 1C**).

### LPS stimulation significantly increased LSK number of CD11a KO mice

To understand how the bone marrow (BM) cells respond to infectious stimuli, we performed LPS stimulation *in vivo*. LSK number significantly increased in CD11a KO mice along with higher proliferation based on Ki67 expression (**Figure 1D**). Only 4.5% of WT LSK cells were Ki67 positive, while around 8% of CD11a KO LSK showed Ki67 expression. In contrast to LSK, CMP number in WT and CD11a KO mice upon LPS injection did not differ (**Figure 1E**). CMP proliferation was much higher than LSK in both WT and CD11a KO mice based on Ki67 expression. In WT mice, 50% of CMP expressed Ki67, while CD11a KO CMPs expressed even slightly more Ki67 than WT counterpart. This explains higher neutrophil counts in CD11a KO mice (**Figure 1F**).

ICAM-1 is a major ligand for CD11a. Thus, we also examined if ICAM-1 KO mice would behave similarly to CD11a KO mice. However, there was no difference in LSK and CMP number between WT and ICAM-1 KO mice following LPS stimulation (**Figure 1G**). Previously, we also examined the role of other β2 integrins CD11b, CD11c and CD11d in HPSCs using CD11b KO, CD11c KO and CD11d KO mice. We found that CD11cKO mice had significantly less LSK cells, but CD11bKO or CD11dKO mice did not have any difference in LSK number compared to WT mice following LPS injection (8). Thus, the expanded LSKs in CD11aKO mice upon LPS stimulation suggests a distinctive biological role of CD11a among β2 integrins.

### Mixed chimera of WT/CD11a KO did not show any difference from WT/WT mixed chimera

**Figure 2:**
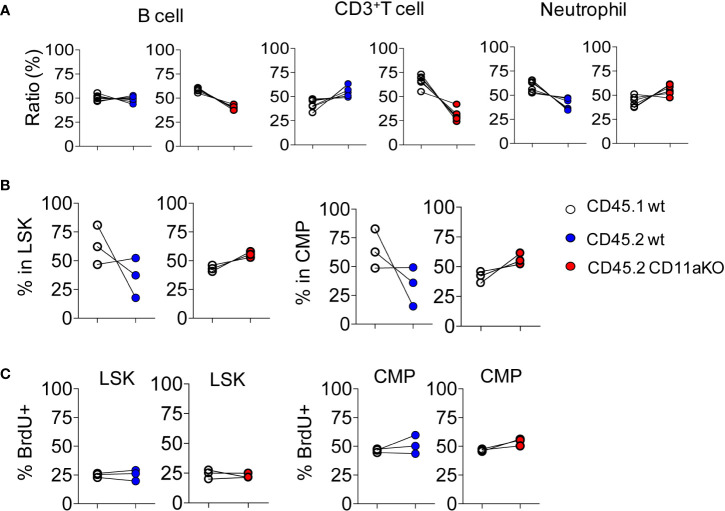
Mixed chimeric mice showed comparable proliferation of HSPCs between genotypes at steady stage. Chimeric mice were made by transplanting the mixture of CD45.1 WT and CD45.2 WT BM cells or CD45.1 WT and CD45.2 CD11a KO BM cells at the ratio of 1:1 into lethally irradiated recipient mice. **(A)** At 8 weeks post-BM transplantation, peripheral blood from recipient mice was collected and subjected to flow cytometry analysis. Ratio was calculated by staining cogenic maker CD45.1 and CD45.2. **(B)**. Ratio of CD45.1 and CD45.2 BM derived LSK and CMP at 8 weeks post-BM transplantation. **(C)**. Proliferation measured by *in vivo* BrdU labeling. The frequency of BrdU^+^ in LSK and CMP in WT/WT chimera or in WT/CD11a KO chimera. Data are one from two independent experiments. Line indicates an individual chimeric mouse.

To investigate whether higher CD11a KO LSK proliferation was intrinsically driven by CD11a, we created mixed chimeric mice by injecting the mixture of bone marrow cells at the ratio of 1 :1 into lethally irradiated recipients. Six weeks later, we monitored the peripheral blood. There was no statistical significance between CD45.1 and CD45.2 cells in both chimeric mice (**Figure 2A**). We then examined their bone marrow cells. There was no statistically significant difference in CD45.1 and CD45.2 derived LSK and CMP (**Figure 2B**). Proliferation was also not different between genotypes (**Figure 2C**).

**Figure 3:**
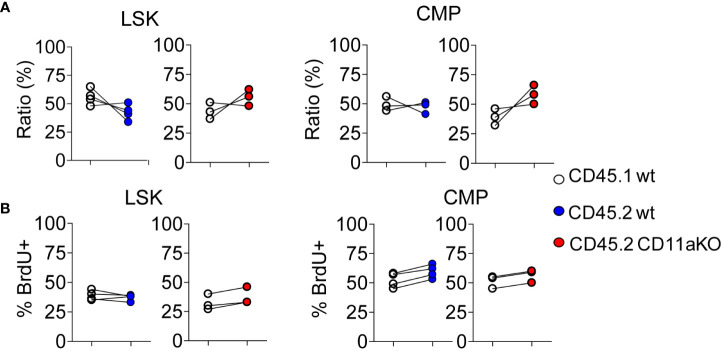
Mixed chimeric mice showed comparable proliferation between genotypes upon LPS injection. Chimeric mice were made by transplanting the mixture of CD45.1 WT and CD45.2 WT BM cells or CD45.1 WT and CD45.2 CD11a KO BM cells at the ratio of 1:1 into lethally irradiated recipient mice. At 8 weeks post BM transplantation, chimeric mice were i.v. injected with LPS, and sacrificed 6 hours later. To measure the proliferation of HSPC, BrdU was *i.v.* injected at 2 hours post LPS injection. **(A)**. Ratio of CD45.1 and CD45.2 BM derived LSK and CMP of chimeric mice post *in vivo* LPS injection. **(B)**. The frequency of BrdU^+^ in LSK and CMP in WT/WT chimera or in WT/CD11a KO chimera post *in vivo* LPS injection. Data are representative from two independent experiments with the same pattern. Line indicates an individual chimeric mouse.

We then examined *in vivo* LPS stimulation in the chimera. We did not find any statistically significant difference in the ratio between CD45.1 and CD45.2 components in LSK and CMP (**Figure 3A**). Furthermore, we did not find any difference in proliferation between genotypes (**Figure 3B**). This result points out that higher proliferation in CD11a KO LSK in CD11a KO mice upon LPS injection was not intrinsically driven. This result was in line with our finding that ICAM-1 KO mice did not show similar phenotype with CD11a KO mice as above.

### CD11a KO mice had elevated IL-27 level after LPS stimulation

**Figure 4:**
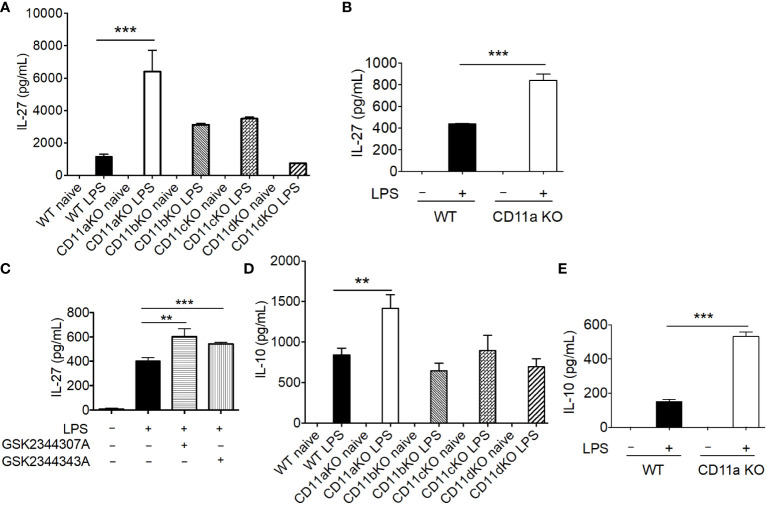
CD11a deficiency leads to enhanced IL-27 production *in vivo* and *in vitro*. **(A)** WT, CD11aKO, CD11bKO, CD11cKO and CD11dKO mice were *i.v.* injected with LPS, and sacrificed 6 hours later. Serum was collected and IL-27 level was measured. N=5 in both groups; Data are one from two independent experiments with the same pattern. B, C and E: *in vitro* bone marrow derived macrophage. **(B)** both WT and CD11aKO bone marrow cells were differentiated into macrophage in the presence of M-CSF, and then were stimulated with LPS for additional 24 hours, and IL-27 in supernatant was measured by ELISA. **(C)** WT bone marrow cells were differentiated into macrophage in the presence of M-CSF, and then were stimulated with LPS for additional 24 hours, in the absence or presence of CD11a inhibitors (10 μM), and IL-27 in supernatant was measured by ELISA. **(D)** Plasma IL-10 level was measure as in **(A)**. **(E)** IL-10 in supernatant was measured as in **(C)**. For **(A, D)** data are one from two independent experiments with the same pattern. For **(B, C, E)** shown are accumulated data from three independent experiments. Statistical analysis was performed using one-way ANOVA with Bonferroni post hoc analysis. **p< 0.01, ***p< 0.001.

Previously, mediators that drive emergency granulopoiesis have been extensively examined in the setting of LPS stimulation, among which IL-27 was shown to expand LSK population (13). Thus, we hypothesized that CD11a KO mice might possess higher IL-27 levels after LPS stimulation. As hypothesized, IL-27 was significantly higher in plasma in CD11a KO mice after LPS stimulation (**Figure 4A**). Plasma from CD11b KO and CD11c KO mice showed a trend that their IL-27 levels were higher than that of WT mice, though statistically not signifincat (**Figure 4A**). IL-27 is primarily produced by myeloid cells (14–16). Therefore, we tested if CD11a KO macrophages produced higher levels of IL-27 *in vitro*, in which case BM derived macrophage (BMDM) was differentiated and used. In line with the finding *in vivo*, we observed that IL-27 level was higher in CD11a KO BMDM stimulated with LPS compared to WT BMDM (**Figure 4B**). We also tested CD11a inhibitors GSK2344307A and GSK2344343A in WT BMDM *in vitro*. These inhibitors directly bind to the ligand binding domain called the I domain in CD11a to block CD11a-ligand binding (12). Similar with CD11a deficiency, both CD11a inhibitory compounds enhanced IL-27 production stimulated by LPS as well (**Figure 4C**). To understand other cytokine profiles, we also measured TNF-α, KC, IL-1β, IL-6, IL-10, IL-12p70 and IFNγ. We found that only IL-10, was significantly elevated in CD11a KO both *in vivo* but not in CD11b, CD11c, and CD11d KO mice (**Figure 4D**). We also found that IL-10 level was higher in CD11a KO macrophages compared to WT macrophages *in vitro* (**Figure 4E**).

## Discussion

In this study, we found that CD11a KO mice had higher LSK proliferation and number following LPS stimulation. Our data showed that this was at least in part due to higher IL-27 production from CD11a KO myeloid cells because IL-27 is a prime driver of LSK proliferation during emergency proliferation. This finding adds a novel role of CD11a although it has been widely recognized a critical adhesion molecule for leukocyte arrest and immunological synapse formation.

Because adhesion is an important feature to keep HSCs in a certain environment, the role of integrins in HSPCs has been previously studied. Integrin α1, α2, α3 are not expressed on HSPCs (17). α4 and α6 are expressed on HSPCs and involved in their homing (18). α5, α7-α11 are also expressed on HSPCs, but their functions for HSPCs are not yet determined (19). Among β2 integrin members, we previously reported that CD11c (αX) regulated the proliferation and cell death of HSPCs (8). CD11c KO HSPCs showed cell death and loss along under LPS stimulation in a cell-intrinsic manner. In contrast, CD11a (αL) KO HPSCs showed an increase in HPSC number following LPS stimulation. However, our result also indicates that CD11a regulated HSPC function not via a cell intrinsic mechanism.

IL-27 is a member of IL-12 and a heterodimeric cytokine mainly produced from antigen presenting cells (APCs) such as macrophages and dendritic cells. IL-27 is rapidly produced by APCs upon exposure to TLR ligands (20). TLR4 stimulation induces IL-27 production by TIR domain containing adaptor inducing IFN-β (TRIF) activation. This leads to the translocation of interferon regulatory factor 3 (IRF3), a critical transcription factor for interferon production. This IRF3 is reported as a master regulator for IL-27 production (21). Previously the crosstalk between CD11b and TLR4 has been described (22). CD11b KO macrophages showed an exaggerated TLR4 signaling responses including TNF-α and IL-1β production, which is the result of TLR4-MyD88 signaling event. So far the interaction between CD11a and TLRs has not been reported yet. CD11a activation induces PI3K activation. It is reported that effector molecule of PI3K-Akt signal suppressed TLR-TRIF signal (23). Thus, CD11a may cross talk with TLR4-TRIF pathway. ICAM-1 is a major ligand for CD11a. However, ICAM-1 KO mice did not show similar phenotype with CD11a KO mice, suggesting that the modulation of IL-27 production by CD11a might not involve its ligand interaction. This topic will be examined in the future.

IL-27 has diverse roles. IL-27 driven emergency granulopoiesis response is an important process to combat against infection (24). IL-27 also promotes proliferation of NK cells and production of IFN-γ, granzyme B and perforin, production of inflammatory cytokines by monocytes, and induces Th1 differentiation (25). In contract, IL-27 also has potent anti-inflammatory effects. IL-27 suppresses Th17 cells in experimental autoimmune encephalitis (26). From infection standpoint, IL-27 can be detrimental (27) or protective (28). Our data supported that CD11a regulates IL-27 production, but we need to examine the role of CD11a-IL-27 response more in depth.

Certainly, it is possible that a factor other than IL-27 is also responsible for our HSPC phenotype. Therefore, it would be interesting to study if there is another factor involved in this phenotype. IL-27-driven transcriptional network regulates IL-10 expression (29). It is also reported that IL-10 regulates progenitor expansion (30, 31). In this study, we also found that CD11a KO mice or macrophages produced more IL-10. Thus, how CD11a regulates these cytokine production network would be an important topic for future investigation.

In summary, we found that CD11a deficiency significantly facilitated HPSCs proliferation under LPS, which was at least in part driven by IL-27 production rather than cell-intrinsic mechanism.

## Data availability statement

The raw data supporting the conclusions of this article will be made available by the authors, without undue reservation.

## Ethics statement

The animal study was reviewed and approved by Institutional Animal Care and Use Committee (IACUC) at Boston Children’s Hospital.

## Author contributions

All authors listed have made a substantial, direct, and intellectual contribution to the work and approved it for publication.
